# Toward an integrative socio-cognitive approach in autism spectrum disorder: NEAR method adaptation—study protocol

**DOI:** 10.3389/fpsyt.2023.940066

**Published:** 2023-05-24

**Authors:** Jihene Bahri, Zeineb S. Abbes, Houda Ben Yahia, Soumeyya Halayem, Selima Jelili, Melek Hajri, Isabelle Amado, Alice Medalia, Asma Bouden

**Affiliations:** ^1^Department of Child and Adolescent Psychiatry, Razi Hospital, Manouba, Tunisia; ^2^Faculty of Medicine of Tunis, University of Tunis El Manar, Tunis, Tunisia; ^3^CH Sainte-Anne, C3RP & Services de Psychiatrie d'adultes HU & 75G17, Paris, France; ^4^Department of Psychiatry, New York State Psychiatric Institute, Columbia University Vagelos College of Physicians and Surgeons, New York, NY, United States

**Keywords:** autism spectrum disoders, cognitive function, social skills, adolescents, cognitive remediation

## Abstract

**Background:**

The cognitive impairments exhibited by people with ASD, threaten the development of social skills that are essential for establishing and maintaining harmonious social relationships. Cognitive remediation and social skills training are now considered as crucial therapeutic approaches in the management of these disorders. Several programs have already been validated and have shown improvements in social skills or cognitive performance. However, the effects of these training methods seem to be difficult to generalize to other everyday life. The aim of our study is to alleviate cognitive and social deficiencies by using a socio-cognitive framework to adapt the Neuropsychological Educational Approach to Remediation (NEAR) method for adolescents with ASD.

**Methods/design:**

Adolescents meeting the DSM-5 criteria for ASD, older than 13 years, and following a regular school curriculum will be recruited from clinical population at the Child and Adolescent Psychiatry in Razi University Hospital-Manouba- Tunisia. Our study is an open and non-randomized controlled trial including 30 patients: NEAR group / control group. The NEAR method combines computerized cognitive exercises and bridging groups inspired from cognitive behavioral therapy. NEAR group will be divided into three groups of five patients each. The duration of the sessions will vary according to the capacities of the participants and the exchanges between them (about 60–120 min). In our study, bridging groups will be amended by adding other tasks including planning role plays and scenarios of problematic social situations in autism, taking into account cultural particularities in order to promote social skills. Computerized exercises will be enriched by adding other tasks aiming to improve the recognition and expression of facial emotions by using digital videos and photographs expressing the six basic emotions. The duration of the program will be about 6 months. All selected patients will have an assessment of cognitive function: social cognition, neurocognition and pragmatic skills, social skills, self-esteem and global functioning at baseline, 1 week after the end of the NEAR program and 6 months later.

**Conclusion:**

This adaptive program is a promising socio-cognitive intervention that create new perspectives for adolescents with autism spectrum disorder.

## 1. Background

Autism spectrum disorder (ASD) is a prevalent childhood neurodevelopmental disorder that affects approximately 0.97% of the population (1). The rising incidence rates have been attributed differently from one country to another to different factors such as increased awareness and greater societal recognition of ASD (2), changes in diagnostic tools and criteria, lowered stigma, and improved health service organization (3).

Although ASD is a very heterogeneous disorder with many associated psychiatric and medical co-occurring conditions, specific core symptoms define its diagnosis. This disorder is largely defined by impaired socio-emotional reciprocity and limited interpersonal communication, a diagnostic criterion in the DSM 5 [Diagnostic Statistical Manual of mental disorders (4)].

The construct of social skills is both multidimensional and highly related to many other important constructs: pragmatic language, cognition, and mental health (5, 6).

Several definitions of social skills agree that these skills are learned behaviors that enable individuals to communicate and interact competently and appropriately with each other, both verbally and non-verbally, in a given social context. This wide group of abilities emerges from the appropriate execution of social cognition processing that allows us to predict and understand other people’s intentions, feelings, emotions, and behaviors (7). They are essential for establishing and maintaining harmonious social relationships as well as for social and professional integration.

People with ASDs have difficulties in acquiring and mastering these competencies. According to clinical studies and descriptions, these difficulties emerge early in a child’s development (poor eye contact, lack of joint attention, lack of anticipatory response, delay in the development of language, receptive language, and symbolic play …) (5, 8, 9). Wing et al. identified three groups of children with ASD: those who were aloof and socially remote, those who moved away from social overtures but could be engaged (passive interaction), and those who engaged in interactions in unusual or odd ways (odd interaction) (10).

Social communication deficits present in ASD are wide-ranging and individuals vary broadly in their clinical presentation. Consequently, impaired social learning in early childhood, differences in the maturation of social behavior continue through developmental cascades (11). Indeed, adolescents with autism frequently struggle with discomfort or ineptitude in social interactions. Their social skills deficits are characterized by pedantic or odd speech patterns, lack of empathy, lack of social problem-solving ability (12), difficulties interpreting body language (13), poor social–emotional reciprocity and non-verbal communication skills impairments (14), difficulties in process facial expressions (15, 16) and impairments in identifying others’ thoughts (17). These difficulties, particularly in adolescence, are the source of traumatic experiences of rejection and misunderstanding in school and social environments. Outside of relational outcomes, Frith (18) further proposed that the social deficits may impact also academic performance and employment outcomes. They are also correlated with a high rate of self-blame and low self-esteem in adolescents with ASD (19). It is no wonder that young persons with ASD are considered particularly vulnerable to developing depressive and anxiety symptoms (20, 21).

In the past decade, there has been significant attention to developing and disseminating information regarding social skills interventions and social skills training that demonstrated mixed outcomes in ASD. In fact, social skills training (SST) which is inspired from cognitive and behavioral interventions and social learning theories (22), is recommended by the French National Authority for Health (Haute Autorité de Santé, or HAS) in ASDs (23). However, fewer studies have included adolescents with ASD. A review of 10 SST intervention studies for youth with ASD, in 2008, showed that seven (70%) of the studies have a positive treatment effect on social skills and significant gains on real-life problem-solving tests (24). In a 2013 Cochrane review evaluating the effects of social skills groups for individuals aged 6–21 with ASD, Reichow et al. (25) showed that there is an improvement in social communication in these groups, friendship quality, and a decrease in feelings of loneliness.

In addition to genetic and neurobiological factors, social dysfunction patterns in ASD are suggested to be primarily underpinned by impairments in cognitive function (26, 27). In our understanding of the underlying cognitive deficit exhibited by people with ASD, psychological theories have been a topic of much debate. They argued that this impairment includes a deficit in theory of mind (ToM) (28, 29), dysexecutive syndrome (30, 31), and a weak central coherence (32). That could explain why aspects of social and communicative behavior that require social insight are impaired in autism. In fact, the adaptation to the immediate social environment requires several high-level cognitive processes that allow the individual to regulate his behavior by taking into account the behavior of others. According to Mottron (33), the social deficits in autism would be explained by the deficit of some basic cognitive functions. These functions are often convoluted and operate synergistically. The notion of weak ‘central coherence’ (CC) in autism has been postulated by 32. It describes a cognitive style rather than deficit (34). In their interaction with their environment, individuals on the autism spectrum disorder tend to excel at focusing on extreme detail and fail to extract global form and meaning. It may be related to ToM because in order to understand the thoughts and emotions of others in a real-life situation, an individual needs to be able to take into account the social context and integrate diverse information from a variety of sources (34).

ToM is one of the most relevant concepts in the field of social cognition, particularly in the case of autism (29). It is defined as the ability to ascribe mental states to oneself and others and to interpret the behavior of others in terms of mental states. It makes it possible to identify the emotion, desires, and thoughts, to understand the communicative intentions of others, and to understand infraverbal communication. It underlies also empathy skills (35). Possessing a functional theory of mind is considered crucial for success in everyday human social interactions. Empirical findings confirm this deficit in mind-reading in ASD based on inferior performance on assessment tasks (mindblindness) (36). A poor ToM will significantly compromise their socially mediated learning.

ToM and the pragmatic dimension of language are also closely intertwined. Several findings from different approaches (mainly neurological and developmental studies) support this perspective (37–39). According to Dardier (40), the pragmatic of language is the “interface between cognitive, social and linguistic developments.”

The major proponents of the concept of “executive function” (EF) have been Russell et al. (41), who argue that basic executive processes underlie the infant’s discovery of his/her own agency. Impairments in ‘Executive Function’ are broadly cited in ASD and have been posited to underlie the core difficulties both in the social and non-social domains. They are essential to guide behavior in a continuously changing environment (42). The theory of dysexecutive syndrome in autism comprises defects in working memory, response inhibition, initiation and monitoring of action, cognitive flexibility, and impulse control (43, 44). Minshew and Goldstein (45) found that individuals with ASD were more impaired than those without autism when the complexity of exercises was increased. These difficulties may clarify some behavior problems such as cognitive rigidity, perseveration to routines, failure in adjusting behavior to changing environmental demands, and stereotypies observed in ASD (46). It is obvious that the executive dysfunction account addresses the social and communicative aspects and non-social aspects of autism.

From this perspective, cognitive deficits are not solely a manifestation of neuropsychological dysfunction, but rather social-cognitive dysfunction (47).

Cognitive remediation (CR), based on the theory of brain plasticity, is now considered a crucial therapeutic approach in the management of cognitive dysfunction. A narrow range of researchers studying the use of CR in ASD has shown improvements in social cognition (48, 49) or executive function (50, 51). A cross-sectional study, by Hajri et al. (52), investigating improvements in executive and academic functioning in 25 patients with ASD after the use of a cognitive remediation program (Cognitive Remediation Therapy), showed a significant improvement in intellectual efficiency, working memory, cognitive flexibility, planning and academic performance.

Currently, there is a wide range of challenges, and paths for fundamental advances in ASD therapeutics that have reported that this population do not readily generalize and maintain skills. Successful generalization in necessary for an intervention to have benefits in everyday life beyond the original learning environment. CR and SST show only moderate effects and transfer into reals world is insufficient. Thus, a combination of CR and skills groups could help.

In view of all these data, we propose to conduct a study aimed at improving the maintenance and generalization of skills to new situations and environments in this population, by adapting the NEAR method. The main objective is to evaluate the effect of cognitive training combined with the NEAR approach (CT-NEAR), delivered in a social rehabilitation framework, on the global functioning of adolescents with autism. The goal is to reduce the negative impact of impaired cognition on the daily life and to enhance of the global functioning.

NEAR, which is used with a variety of cognitively challenged psychiatric populations, includes computer based cognitive exercises and verbal “Bridging groups” to help patients transfer their practice of cognitive and social skills with real-world situations. Adolescents with ASD require more of a focus on social cognitive skills which is typically provided in the NEAR program. Our adaptation work aims to provide, through these skill-building groups, an opportunity to improve social skills as well as other competencies. Thus, the adaptations would increase focus on improving language pragmatics, ToM, empathy, and attributional style. These adapted “Bridging groups” would also aim to increase the effectiveness of the therapeutic process by encouraging the process of generalization that promotes the application of cognitive skills and compensatory strategies acquired in the sessions to situations outside of the group (53).

## 2. Methods

### 2.1. Interventions

#### 2.1.1. Method of choice: neuropsychological educational approach to remediation (NEAR)

This holistic approach to cognitive remediation, developed and disseminated by Medalia and colleagues, was specifically designed for use with psychiatric patients (47, 53). NEAR aims to improve cognitive functions that have been identified as sufficiently impaired to hamper functional outcome. This method combines computerized cognitive exercises and bridging groups inspired from cognitive behavioral therapy. It promotes a smooth interplay of cognitive and social–emotional variables in everyday functioning. NEAR has a specific focus on motivation which has benefits on learning and is also associated with increased levels of autonomy, self-esteem, and positive experiences (54). This determinant is very important for young patients. Further, this rehabilitation program promotes awareness about learning style, learning strengths, and weaknesses so participants gain a sense of competence and confidence in their ability to acquire skills (47).

The NEAR model was designed to be easily implemented in a variety of settings and cultures, which has facilitated worldwide dissemination and the translation of materials into multiple languages. In France, there has been a large-scale adoption of the NEAR program, and clinician training and patient materials have been translated into French, which provides a ready source of materials for use with the French speaking Tunisian population. As implemented throughout France (55), participants are seen in groups of 4–8, with each having their own computer to work on. The duration of a clinician led session lasts 90 min, with 45 min reserved for computerized exercises and 45 min for the bridging group. The role of the clinician in NEAR sessions varies from instructing to assessing, to observing, to facilitating a positive learning experience by encouraging participants to seek and find strategies. Manuals of bridging group discussions are available to enable clinicians to relate activities of daily life to the cognitive skills worked during the sessions. Worksheets and a variety of forms are provided in the clinician manual (53).

The computer-based exercises to be performed during each session are personalized to each participant’s cognitive needs, as identified by a neurocognitive evaluation and interview. Thus, participants are seen in a group but work on different exercises personalized to their needs. These exercises may target several cognitive domains such as visual and auditory attention, focused and divided attention, sustained attention, processing speed, verbal and visual working and long-term memory, planning, problem-solving, inhibition, and mental flexibility. The repetition of the exercises and the presentation of the results and the level of progress on the screen of each participant enable the reinforcement of autonomy and favor the use of errorless learning. Participants conclude their computer based cognitive activities for the day by completing a worksheet about the exercises completed, the level reached, the cognitive function worked on, the link with daily life. Further comments may pertain to the strategy used in each exercise, the benefit(s) of the session, and the difficulties encountered. These activities encourage self-reflection, metacognition and enhanced cognitive skillfulness to handle everyday situations.

The Bridging session manual used in France covers 34 sessions organized in 8 modules (Table 1).

**Table 1:** The NEAR France program modules.

	Title	Number of sessions
Module 1	Introduction to cognitive skills	7
Module 2	Verbal reasoning and problem solving	3
Module 3	Thinking about your own thoughts	5
Module 4	Planning and organization	4
Module 5	Attention	4
Module 6	Effective communication	8
Module 7	Healthy living	2
Module 8	Conclusion	1

#### 2.1.2. Adaptation of NEAR method to adolescents with ASD

In our study, the 6th Module (Effective Communication) will be enriched and amended by adding other tasks aiming to improve the development of non-verbal communication, conversational skills, assertiveness, social skills in group situations, and emotional recognition. We will also add a new session entitled “Social rules and respect for others” (two parts). This module will then include 8 sessions. The sessions of this module are structured around a first theoretical part. The second part consists of an application in the ecological environment (practical exercises carried out in small groups). The approach through the different sessions is progressive, allowing to reach the most complex objectives related to social autonomy.

According to the theme of the session, the moderator will choose 3 or even 4 of the following functions per session: Verbal and non-verbal theory of mind/ Facial emotion recognition /Empathy/ Recognition of simple and complex emotions/ Recognition of automatic thoughts (reinforcement: this topic was addressed in module 3)/Attributional styles/ Pragmatics of language.

Topics will include problematic situations for adolescents with ASD: two-way conversation with a peer, teacher…; sharing information with peers; understanding humor, teasing, innuendo, and metaphors; communication through messaging and social networks; learning social rules; respect for others, recognizing one’s limits in an interaction; organizing meetings/outings with peers; management of friendships and romantic relationships; management of stress caused by school learning and bullying; handling disagreements and arguments.

Tools that will be used in this module are:

➢ Scenarios of problematic social situations in autism, taking into account cultural particularities (Theory of Mind). We created a list of social story ideas that include common social situations that autistic children might encounter to help them to cope with various changes and everyday life transitions. Those situations, allow them to have a better comprehension of their behavior as well as others’ and provide tools that aim to teach them how to make and maintain friendships, join group activities, initiate friendships and social relationships as well as maintain them. The use of these social narratives aims also to teach them how to ask for help and get unstuck.
Role-playing exercises with peers in order to enhance participation in the community and support outcomes like self-esteem and friendships. Role-plays are preceded by the direct instruction of skill so that adolescents have a foundation on which to then practice the skill.
➢ Digital videos and photographs expressing the six basic emotions (happiness, anger, sadness, disgust, fear, and surprise), presented by actors of different ages and genders, to improve the recognition and expression of facial emotions. The items are coded according to Ekman’s “Facial Action Coding System” method. During this task, each facial expression with the respective proposals remains on the screen for 15 s. After this time and when the participant has not been able to select his choice, the correct answer will be displayed on the screen. Once an answer is selected for a given face, the response time will be recorded and the correct answer will be displayed on the screen.
➢ Mime of emotions to improve emotional skills through facial expression recognition. During this task, the participant takes it in turns to take a card and mime the emotion while the others try to guess. Then, each candidate talks about an event for which he experienced this emotion.
➢ “Feelings” Game: a board game created in 2015 by Jean-Louis Roubira and Vincent Bidault (56). This game aims at expression and empathic exchange through emotions. “Feelings allows you to learn more about yourself and the other players, to be open to all the ways in which we are different or the same, and to consider everyone as a unique person,” Thibaut Quintens (Act in games). Around the Emotions Track placed in the middle of the table and after reading a given situation (120 Situation cards divided into 3 themes, family, friends, and school), each player is invited to position himself on one of the proposed emotions (24 Emotions cards) and which he feels closest to. Then, persons exchange glances with their playing partner and place a bet on the emotion they think their partner has chosen (72 Vote cards, of different colors, 9 per player). Each player selects from his Vote cards the one he thinks corresponds to the personal emotion of his partner. Each player selects from his Vote cards the one he thinks corresponds to the personal emotion of his partner. Each player guesses the feeling chosen by their teammate. When both players guess the right feeling, they earn three points. If just one of them guesses the right feeling, they earn one point, and if neither of them guesses they have no point. The aim is to find the emotions of the other person, the track of emotions will then reflect the degree of empathy and openness to others. ‘Feelings’ can be used as a trigger to discuss feelings in specific situations (56, 57).
➢ Team Building Activities: group discussion based on free associations of ideas (using a dashboard), discussions around short videos (using a humorous Tunisian television series) to predict a character’s behavior based on his or her mental state, telling a story to their teammate.
➢ Home tasks, at the end of each session, to promote generalization and spontaneity (transferring skills into natural environments and everyday interactions): introduce themselves and get leadership on making new friends, discuss their interests with others and suggest a group game, plan an outing with your friends.

### 2.2. Study design

Our study is a prospective, experimental, open, and non-randomized controlled trial.

This study research is carried out in the Child and Adolescent Psychiatry Department in Razi University Hospital-Manouba-Tunisia. It is conducted in collaboration with the C3RP team (Resource Center for Cognitive Remediation and Psychosocial Rehabilitation, Ile-de-France) -Sainte-Anne Hospital and under the supervision of Dr. Alice Medalia (Ph.D. – Professor of Psychology at Columbia University Irving Medical Center and Director of Cognitive Health Services for the New York State Office of Mental Health, United States).

### 2.3. Participants and screening

The inclusion criteria are:

ο Adolescents aged than 13 years, meeting the Diagnostic and Statistical Manual for Mental Disorders (DSM-5) (4) criteria for ASD confirmed by the ADI-R [Autism Diagnostic Interview-Revised, (58)] criteria, without mental retardation, followed in the Child and Adolescent Psychiatry Department (out-patient clinic) in Razi University Hospital-Manouba-Tunisia. Patients who agreed to participate in our study were retained.
ο Sufficient level of understanding and communication in Arabic and French (allowing them to understand the instructions during the care sessions).
ο Regular school curriculum.

It should be noted that adolescents treated with psychotropics will be included, with the condition that there will be no change to the drug treatment (molecule, dosage) during the period of study.

A certified therapist will conduct a standardized interview for the clinical and diagnostic evaluation of these adolescents (using the ADI-R).

Parents obtained information concerning the study and had the opportunity to ask questions before agreeing to the participation of their children, and during the study.

The non-inclusion criteria are:

ο Out-of-school patients
ο Mental retardation or severe cognitive impairments
ο A history of neurological disorder
ο Severe behavioral disorders
ο Electroconvulsive therapy in the past 6 months

Exclusion criteria are:

ο Non-achievement of the program
ο Missing more than three sessions
ο Non-achievement of assessments after finishing the therapy

Strategies to improve adherence to intervention:

ο Method of choice: NEAR program enhances intrinsic and extrinsic motivation by employing more engaging and interesting exercises packages for cognitive practice, involving participants in choosing the focus of training.
ο All candidates received two psycho-education sessions: one individual and one group session in order to raise their awareness, motivation and to present the program (number of sessions, progress, interest…).
ο Parent involvement in the program and home tasks.

Our study will include 30 patients that will be divided into two groups: NEAR group/ control group (a group that will assign usual care).

This allocation of patients between the two groups would be made according to the following criteria:

ο Teenage motivation
ο Their availability and parent’s one to accompany them to their clinical evaluations and therapeutic sessions.

The patients included in the NEAR group will be divided into three groups of five patients each. The rhythm for sessions for each group is one to two sessions per week. The duration of the program will be about 6 months.

In our study, the duration of the sessions will vary according to the capacities of the participants and the exchanges between them (about 90–120 min).

Two pre-trained therapists lead the sessions and are assigned to follow the progress of each participant.

All selected patients will have an assessment of global functioning, cognitive function (social cognition and neurocognition) pragmatic skills, social skills and self-esteem, and at baseline (T1), 1 week after the end of the NEAR program (T2), and 6 months later (T3). The same interval will be kept for three sessions of evaluations of the control group.

### 2.4. Assessments

#### 2.4.1. Global functioning

ο Global Assessment of Functioning (GAF):

In our study, the GAF is used to evaluate subjectively the social and psychological functioning of the participants and the impact of psychiatric illness on an individual’s life and daily functional skills and abilities. Scores range from 100 (“extremely high functioning”) to 1 (“severely impaired”). It differentiates 10 levels of psychosocial functioning (59). A higher score reflects higher functioning in everyday life based on scored criteria.

ο Behavior Rating Inventory of Executive Function (BRIEF):

Executive function behaviors in the school and home environments will be assessed using the BRIEF. This questionnaire is elaborated for parents and teachers of school-age children. It includes eight clinical scales (Behavioral regulation scales and Metacognition scales) and two validity scales (Inconsistency and Negativity) that give the clinician a well-rounded picture of the behavior of the adolescent being rated. The Global Executive Composite score takes into account all of the clinical scales and represents the adolescent’s overall executive function (60).

#### 2.4.2. Social cognition

We will evaluate social cognition using the Tunisian Social Situations Instrument (TSSI) (61) and the Facial Emotions Tunisian Test for Children (FETTC) (62). These tasks are Tunisian validated and computerized tests. They consist on downloadable applications on Android (more convenient and more attractive for patients).

ο Tunisian Social Situations Instrument (TSSI) (61):

It is a comprehension test composed of 10 social situations evaluating the attribution of intentions and epistemic and affective mental states to the protagonists of the social stories. Each situation includes a text written in the Tunisian Arabic dialect illustrated by one or more pictures with a synchronized reading of the text and questions. The reading of the texts and the questions is done automatically. The examiner inputs the participant’s answers on the android device. The overall score ranges from 0 to 25.

The social situations were inspired from The Faux Pas test (62), Sally and Anne test in its original (63), the revised version suggested by Riviere in a personal communication (62) and the strange stories of (64).

ο Facial Emotions Tunisian Test for Children (FETTC) (62):

The test consists of a combination of a static and a dynamic subtest. The static subtest includes 114 photographs of actors mimicking six basic emotions (happiness, disgust, fear, surprise, sadness, and anger) and neutral expression with three levels of intensity of facial emotions for the six basic emotions: low, medium, and high. The dynamic subtest includes 36 videos with actors mimicking six basic emotions. Participants are asked to view each face and to identify and select the facial emotion that corresponds to each photo or video.

During the task, each proposed facial expression with the proposals remains on the screen for 15 s. After this time and when the participant was not able to select his choice, the next stimuli were presented. Once a response is chosen for a given face, the participant is not able to revise it, and the next proposal of facial emotion is presented.

#### 2.4.3. Neurocognition

The following table summarizes the tests used to assess the different cognitive functions (Table 2).

**Table 2:** Tests used to assess neurocognition.

Cognitive function to be assessed	Test	Comments
Intellectual efficiencies	Raven’s Progressive Matrices (SPM)	It assesses general non-verbal mental abilities and deductive reasoning/A non-verbal estimate of fluid intelligence (63)
Processing speed	Wechsler Adult Intelligence Scale III (WAIS III)/Wechsler Intelligence Scale for Children (WISC-5)	The test includes 15 subtests that provide an extensive assessment of both verbal and non-verbal abilities. The Processing Speed Index (PSI) included Digit Symbol-Coding (DSC) and Symbol Search (64)
Working memory and selective attention	The digit-span task	Digit Span Forward+ Digit Span Backwards (65)
	Baddeley’s double task	It includes three steps: Single-task condition/Tracking-Single-task condition/Dual-task condition (66)
Episodic memory	Grober and Buschke’s task	Adapted to the Tunisian population in 1998. It encompasses several phases: a control-encoding task, three free recalls for all items and three cued recalls for items not retrieved at free recall, a recognition phase, and a delayed free/cued recall phase (after 20 min) (67).
Executive functions	Trail Making Test A and B (TMT A and TMT B)	TMT A evaluates attention. TMT B evaluates inhibition, mental flexibility, and working memory (68).
	STROOP test	It measures the ability to inhibit cognitive interference (the Stroop Effect), selective attention capacity and skills, and processing speed (69, 70).
Visuo-constructional ability	Rey Complex Figure (RCF)	It is used for the assessment of visuo-constructional ability, planning, and visual memory (71).
Cognitive flexibility	Verbal and semantic Fluences Tests	We will use the Arabic version. It consists of 3 parts: The phonemic fluency task (using the initials “ق” then “ب”), the semantic fluency (animals then fruits), and the alternate verbal fluency (by alternating a word belonging to the clothing category with a word beginning with the letter “ل”) (72, 73).

#### 2.4.4. Pragmatic skills

The Children’s Communication Checklist (CCC-2) Bishop (74), will be used to identify and evaluate pragmatic language impairment in this population. It is a parent or caregiver-rated questionnaire that quantifies the strengths and weaknesses of children’s communication from 0 (“less than once a week”) to 3 (“several times a day”). It includes 70 items on 10 pragmatically constructed subscales: Speech, Syntax, Semantics, Inappropriate Initiation, Coherence, Use of Context, Non-verbal communication, Social Behavior, Stereotyped language, and Interests (75). The CCC reports a sensitivity value of 0.89 and a specificity value of 0.97 for identifying children with autistic symptomatology and pragmatic social impairment (76).

#### 2.4.5. Social skills

The Social Responsiveness Scale (SRS) (77) is a validated questionnaire for assessing the ability to engage in emotionally appropriate reciprocal social interactions, communication, and stereotypies in autism. The scale is filled out by parents and measures the degree of social impairment in participants. Five subscales are distinguished: awareness of the relational environment, social cognition, social communication, social motivation, and mannerisms. It comprises 65 items on a 4-point Likert scale generating one total score (max. 195). A coefficient of social reciprocity is calculated by combining these different subscales. High scores are associated with more severe social impairments. The SRS scores correlate strongly with algorithm scores of the ADI-R (78).

#### 2.4.6. Self-esteem

The Rosenberg Self-Esteem Scale (RSES) (79) is a 10-item scale that measures global self-worth or self-acceptance with both positive and negative feelings about the self. All items are answered using a 4-point Likert scale ranging from “strongly agree” to “strongly disagree.” Higher scores indicate higher self-esteem.

#### 2.4.7. Study procedure

**Figure 1:**
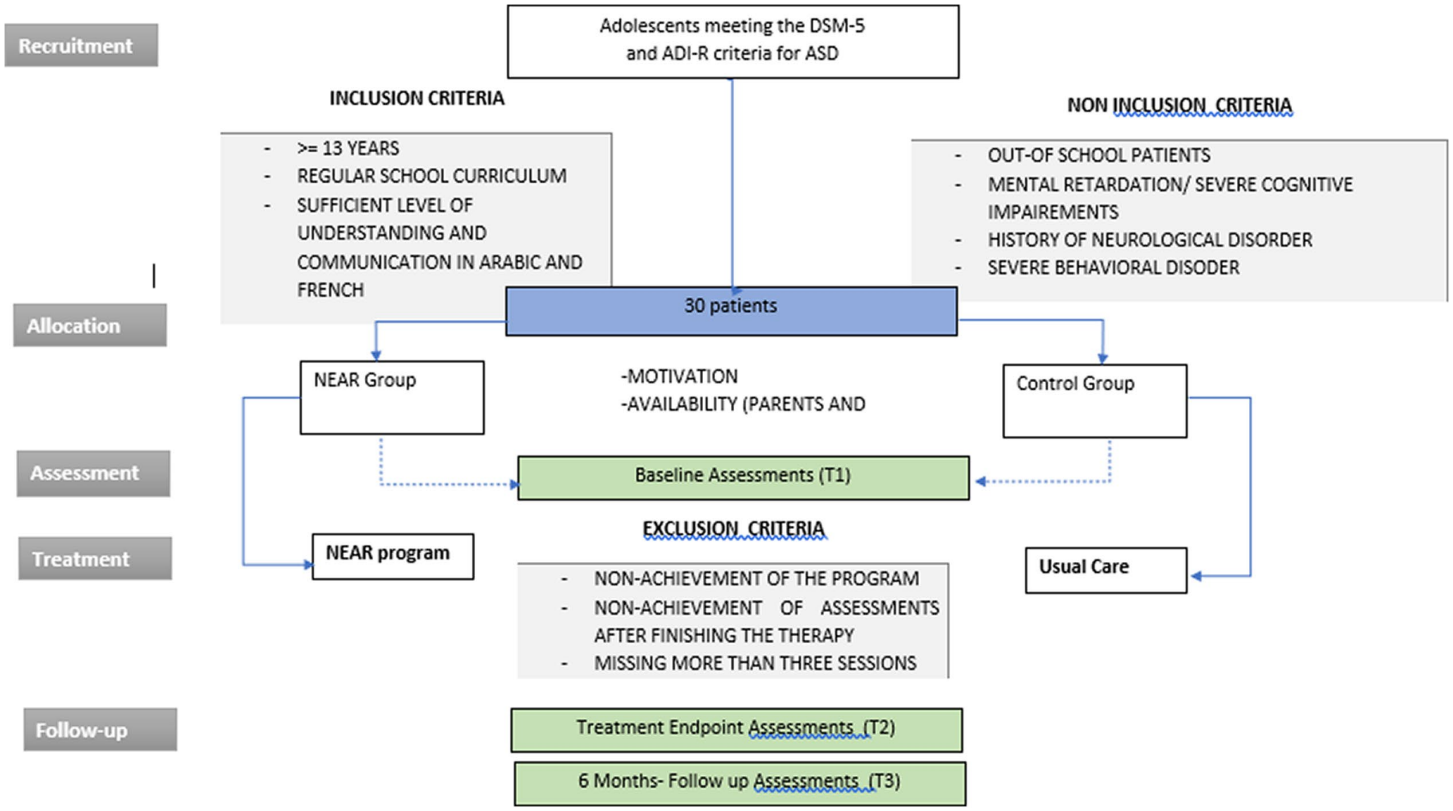
Study procedure.

The study procedure is represented in the Figure 1.

## 3. Statistical analysis

Data collection and statistical analysis will be performed using the Statistical Package for Social Sciences (SPSS) version 25.

We will proceed to the comparison of the different scores of each group at the beginning and at the end of the study as well as to the comparison of the progression of the two groups between them at the beginning and at the end of the study.

The primary objective of our study is to facilitate the transfer of new skills to daily life and thus the global functioning of this population. Primary outcomes will be based on the GAF score and The Global Executive Composite score of the BRIEF. All other measures (social cognition, neurocognition, social skills, pragmatic skills and self-esteem) will be secondary outcomes and will be assessed descriptively.

For the descriptive statistics, we will calculate means and standard deviations for age and educational level and for categorical data, e.g., sex we will use Fisher’s Exact Chi-squared test.

For each neurocognitive and social cognition domain measure, we will conduct *t*-tests for independent samples to examine baseline differences for this sample. To examine Group X Time interaction for measuring treatment effects, we will conduct a Repeated Measure-Analysis of Variance (RM-ANOVA) that compared the difference scores of NEAR-group vs. Control-group at baseline and at the completion of the intervention.

A RM-ANOVA will also be used to examine differences in pragmatic skills, social skills and self-esteem.

For the two measures where there were statistically significant baseline differences, we will repeat the RM-ANOVA using baseline cognitive scores as a co-variate to control for baseline differences.

The significance level for all statistical analyses will be set to *p* = <0.05.

## 4. Ethical considerations

ο Informed consent of the parent has been obtained.
ο Information form will contain: Justification for research, the outline of the study, risks, confidentiality, and voluntary participation telling patients about the freedom to withdraw from the study whenever they wish to (without any change in their usual treatment).
ο Confidentiality indicates how the personal information obtained from the patient will be kept secret (Data safety).
ο Ethical committee approval has been obtained.

## 5. Discussion

The NEAR program is tailored to the participant’s age and interests. It will address the adolescent’s behavior, cognitive and communication skills and offer regular reinforcement of positive actions. Also, parents as well as other family members, will be involved in the treatment program (through home tasks) so that it becomes part of the adolescent’s daily life.

At the end of our study, we expect to see an improvement in terms of social functioning and social skills in this population and so a better social integration and quality of life. Thus, this adaptative program could be a promising socio-cognitive intervention that creates new perspective for adolescents with ASD. In fact, the social demands of adolescence present a particularly difficult developmental stage and a continuous challenge throughout the life span, particularly for people with ASD. Despite their social impairments, maintaining and sustaining friendships remained the greatest challenge for adolescents with ASD and they often express concerns about their lack of reciprocal friendships (80).

Also, we expect neuropsychological improvements, mainly in theory of mind (ToM), which is one of the most frequent and disabling cognitive impairment in autism (28, 81). This improvement could be helped by the enrichment added in bridging groups with scenarios of problematic social situations in autism. This will promote the development of social coping skills, as well as to work through behavior management strategies. In fact, social stories can be effective for individuals with ASD and provide a framework for understanding and addressing ToM deficits characteristic of these individuals (82–84). The choice of group role-plays is motivated by the results of many studies that discuss the use of this method to develop social and pragmatic skills of autistic children (85, 86). Furthermore, “Feelings” game will be used to enhance empathy in our population. It uses two game mechanics to generate empathy: naming which emotion is felt in a particular situation (Introspection) and guessing which feeling other people feel in the same situation (decentering) (56). It is important to highlight that people with autism have a deficit of cognitive empathy but a surfeit of emotional empathy (87).

In this study, the experimental group will benefit from cognitive remediation with NEAR, which combines cognitive remediation and social rehabilitation. This program targets a wide range of neuropsychological deficits (attention, visual field, verbal and visual short-term memory, working and long-term memory, planning, problem-solving, inhibition, mental flexibility…) with an expectation that the improvement after program will translate into greater competency in daily life (47, 88).

NEAR method focuses also on adhering to principles of motivation and learning. The impact of motivational factors on the development of social skills and social cognition in ASD are thought to have downstream effects on the development of social cognition (11). Moreover, this method represents an attractive therapeutic tool for young subjects. In a pilot study testing the NEAR program with four preadolescents and adolescents aged 11–15 years with either ADHD or ASD, all participants enjoyed the program. Also, they found it a fun and effective way to learn new strategies, and they found a pleasure of seeing each other again every week. They perceived support and the development of self-help skills (55). NEAR method also integrates the metacognitive dimension that helps the participants to develop the capacity to reflect on their own cognitive processes. Some studies suggest that metacognitive aspects are of particular importance for social abilities in children and adolescents with ASD (89). The sharing time during the Bridging group (difficulties encountered during the exercises, strategies used, link with daily life, …) aims to foster self-esteem, reinforce intrinsic motivation but also to improve their autonomy, social functioning of the participants, and improve pragmatic skills.

From this same perspective, the impact of cognitive remediation on functional outcomes appeared to be significantly greater in studies that also included psychiatric rehabilitation, suggesting that these two therapeutic approaches may act synergistically (90, 91).

Taking into account the particularities of this population, some difficulties may be encountered by the therapist and arise in performing this study. The reasons relate to some of the characteristics of ASD. The first issue is that in a group situation, the clinician may struggle to accommodate their unique learning styles and needs. In fact, the heterogeneity of symptoms, the presence of behavioral disorders, the lack of reciprocity and autonomy represent a challenge for workers in medico-social institutions (92, 93). Additional limitation that the therapist may face during the bridging group is the characteristics of autistic speech which may differ significantly from one person to another: participants may say things that have no meaning or that do not relate to the conversations they are having with others (off-topic subject), they may repeat words he or she has heard (Immediate echolalia and delayed echolalia), they may ask rhetorical questions, they may have difficulties in understanding idiomatic expressions and metaphors… Therefore, the duration of the sessions will vary according to the capacities of the participants and the exchanges between them and the therapist has to adjust to the different situations encountered.

Moreover, adolescents with ASD, having narrow interests can widely interfere with the different activities of this adapted program. This lasting, intense interest is a common behavior in children with Autism, according to the National Institute of Health (NIH) (up to 88%) (94). In these cases, instead of attempting to prevent or continuously redirect the participant from talking about a preferred interest, the therapist will try incorporating this interest to help the candidate engage in conversation with others and so encourage him to become more socially engaged with the rest of the group. Once the conversation is going, this can be a great opportunity for the therapist to help them learn to take another person’s point of view that would create a positive organizational climate in the group.

Besides, anxiety and autism are often closely intertwined, this comorbidity is linked with significant functional impairment, and it can be related to a neurological response. Specific overlapping areas of the brain are likely involved in both the manifestation of autism and anxiety. For example, neural responses in the brain related to how a person processes rewards can be a risk factor for anxiety and a risk factor for autism (95). This anxiety may be manifested in intolerance and fear of change and uncertainty during the sessions and frustration at difficulty or failure in computerized cognitive exercises and so a quick change of exercises which can affect the effectiveness of the cognitive training. Finally, pharmacological therapy could have an effect on cognition and the chance to NEAR program response.

## 6. Expected outcomes

A better cognitive status, improvement of social skills and pragmatic skills, improvement of self-acceptance, self-esteem and motivation of the person for his/her own change, better quality of life of the patient with autism, thus will lead to a better psychosocial rehabilitation.

## 7. Trial status

The recruitment of subjects began in July 2021 and still continued. Twenty-six subjects were recruited so far. Fifteen adolescents benefit from cognitive remediation with the NEAR up to now.

## Data availability statement

The original contributions presented in the study are included in the article/supplementary material, further inquiries can be directed to the corresponding author.

## Ethics statement

The studies involving human participants were reviewed and approved by the Ethical committee Razi University Hospital-Manouba- Tunisia. Written informed consent to participate in this study was provided by the participants’ legal guardian/next of kin.

## Author contributions

JB, ZA, HB, MH, SJ, IA, AM, and AB contributed to conception and design of the study protocol. JB, ZA, and HB contributed to the assessment of social cognition, neurocognition, pragmatic skills, social skills, self-esteem and global functioning of all the participants. JB, ZA, and HB supervised the conduct of NEAR sessions. IA, AM, and AB contributed to manuscript revision, read, and approved the submitted version. All authors contributed to the article and approved the submitted version.

## Conflict of interest

The authors declare that the research was conducted in the absence of any commercial or financial relationships that could be construed as a potential conflict of interest.

## Publisher’s note

All claims expressed in this article are solely those of the authors and do not necessarily represent those of their affiliated organizations, or those of the publisher, the editors and the reviewers. Any product that may be evaluated in this article, or claim that may be made by its manufacturer, is not guaranteed or endorsed by the publisher.

## Abbreviations

ASD: Autism Spectrum Disorder
NEAR: Neuropsychological Educational Approach to Remediation
SST: social skills training
ToM: theory of mind
CR: Cognitive remediation
EF: Executive Functions
TSSI: Tunisian Social Situations Instrument
FETTC: Facial Emotions Tunisian Test for Children
